# Extracorporeal membrane oxygenation cannula-related chronic wound with a retained suture in a neonate

**DOI:** 10.1093/jscr/rjaa593

**Published:** 2021-02-15

**Authors:** Inez Eiben, Paola Eiben, Mobinula Syed

**Affiliations:** Department of Plastic Surgery, Guy’s and St Thomas’ Hospital NHS Foundation Trust, Westminster Bridge Road, SE1 7EH London, UK; Department of Anaesthesia, Barking, Havering and Redbridge University Hospitals NHS Trust, Rom Valley Way, RM7 0AG London, UK; Department of Plastic Surgery, Guy’s and St Thomas’ Hospital NHS Foundation Trust, Westminster Bridge Road, SE1 7EH London, UK

## Abstract

Extracorporeal membrane oxygenation (ECMO) is often employed to manage persistent pulmonary hypertension of the newborn where non-invasive therapies have failed. Delivery of ECMO requires insertion of indwelling catheters into central or peripheral vasculature; and this predisposes the recipient to development of catheter-associated skin infection; however, chronic non-healing wounds with granuloma formation are rare. We describe a case of an 8-month-old child who presented to our Plastic Surgery Services with a chronic left groin wound at ECMO cannula insertion site that failed medical management. The patient underwent wound exploration and debridement during which an old non-absorbable suture localized at the base of the wound was discovered. The foreign material as well as granuloma was removed, leading to the resolution of the chronic skin lesion and patient recovery without major complications. To our knowledge, this is the first case report describing an ECMO-associated paediatric chronic wound.

## INTRODUCTION

Persistent pulmonary hypertension of the newborn (PPHN) occurs as a result of failed circulatory transition at birth that can be idiopathic or acquired. The most common pathology associated with PPHN is meconium aspiration syndrome (MAS) [1]. Initial treatment strategies include supportive measures involving intubation and gentle ventilation with recruitment manoeuvers and pharmacological interventions aimed at pulmonary vasodilatation, such as nitric oxide or sildenafil. If these methods fail, extracorporeal membrane oxygenation (ECMO) is recommended as a rescue therapy [2].

ECMO is an advanced life sustaining measure that provides temporary respiratory and cardiac support by establishing a circulatory bypass. Two types of ECMO circulations exist: veno-arterial and veno-venous (VV), providing cardiopulmonary and pulmonary support, respectively. Delivery of ECMO relies on the introduction of large-bore cannulas into central or peripheral vessels that allow cycling of blood through the device; for VV ECMO, usual access sites include the jugular vein as inflow and femoral vein as outflow tracts. Non-absorbable sutures are normally used to firmly secure the catheters to theskin.

While complications associated with vascular insertion such as infection, soft tissue damage, distal ischaemia and pseudoaneurysm formation are well recognized, they are relatively rare [3–5]. Moreover, chronic non-healing wounds are not normally associated with ECMO. In this article, we describe a case of ECMO-induced chronic non-healing wound in an infant where foreign material in the form of a suture was found embedded deep in the wound.

## CASE REPORT

A Caucasian female was born at 41-week gestation by emergency lower segment Caesarian section weighing 3.6 kg. Postnatal adaptation was severely decreased with Apgar score of 1 out of 10 at 1 min. The neonate required resuscitation and intubation for respiratory insufficiency. During airway management procedures, meconium presence was noted around and below the vocal cords resulting in a diagnosis of MAS. Despite initial management, the neonate’s respiratory function did not improve and she required a transfer to a specialist paediatric centre where ECMO was commenced on Day 2 of life. Uneventful insertion and removal of ECMO cannulas was documented with the right internal jugular (RIJ) vein and the right femoral vein being the inflow and outflow access sites, respectively. The patient was decannulated after 3 days. Post-ECMO recovery period was complicated by the development of occlusive thrombi in both access vessels, which required therapeutic anticoagulation and vein ligation. Ultrasound imaging of the vasculature revealed an absent RIJ vein, with patent blood flow in collateral vessels and an absent common femoralvein.

**Figure 1:**
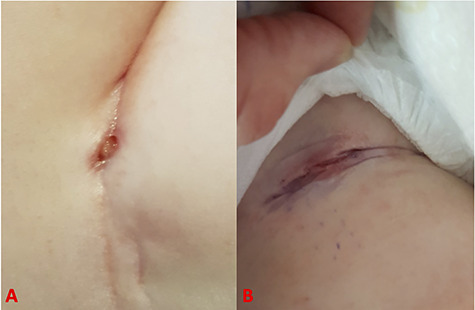
Pictures showing the appearances of the groin wound pre-surgical debridement at 3 months (**A**) and 9 months (**B**) after decannulation.

**Figure 2:**
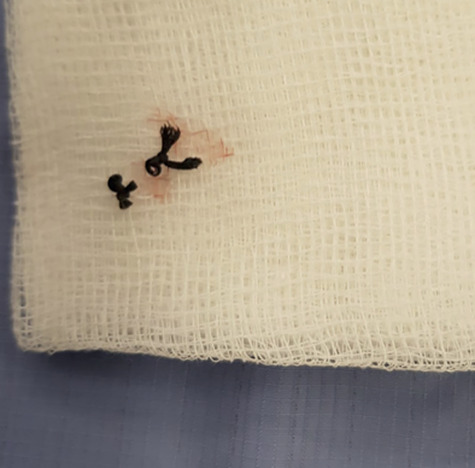
A photo of the suture material found at the base of the wound.

**Figure 3:**
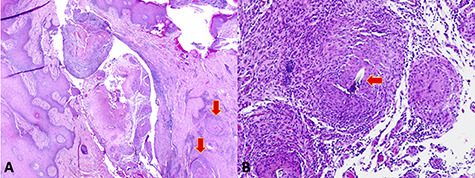
(**A**) Low-power microscopy image demonstrating a skin tract containing necrotic material and surrounding granulomas (arrows). (**B**) High-power microscopy image showing suture material embedded in the granulomatous tissue (arrow).

**Figure 4:**
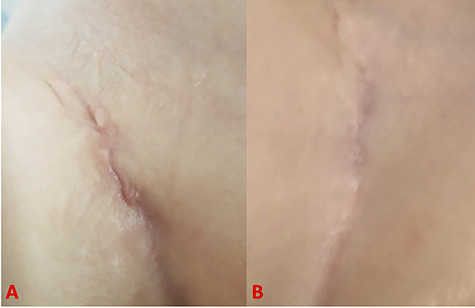
Pictures showing wound healing process at 5 days (**A**) and 4 months (**B**) after surgery.

Although the child continued making good systemic recovery, and despite attempts to manage the wound with antibiotics and a variety of different dressings by the tissue viability team, the wound in the right groin did not heal. As a result, at 8 months of age, the patient was referred to the Plastic Surgery Services in our hospital, where an assessment uncovered a chronic non-infected wound (Fig. 1). Ultrasound investigation suggested a possible foreign body and/or collection. Consequently, the patient underwent elective wound exploration and debridement where a retained suture (Fig. 2), with an associated granuloma (Fig. 3) at the base of the wound, was discovered. This was excised and the wound was closed primarily. The patient was discharged the same day with good prognosis. Follow-up confirmed good wound healing with formation of aesthetically pleasing scar (Fig. 4).

## DISCUSSION

Chronic wounds are defined as wounds that have failed to progress through an orderly and timely process of healing to produce anatomical and functional integrity [6]. Three subsequent but overlapping stages of wound healing are generally recognized: inflammatory, proliferative and maturation/remodelling. During the inflammatory phase, the wound is cleared of pathogens and foreign material by the action of phagocytes and a platelet-mediated fibrin scaffold is formed. The proliferative phase occurs between Days 4 and 21 post-insult and centres around fibroblast-mediated stabilization with collagen and glycosaminoglycans. This is followed by re-epithelialization, neovascularization and tissue granulation. Subsequently, maturation and remodelling occur [7].

Chronic wounds can arise due to a dysregulation at any stage of normal healing [8]. Additionally, localized and/or systemic factors including retained foreign material, circulatory impairment, hypoxia or abnormal inflammatory processes can play a role. Specifically, in the case of foreign body granulomas, the predominant pathological mechanism is generally thought to be an aberrant immune response: due to the presence of foreign tissue that cannot be expelled, the body continues mobilizing an immune response, resulting in persistent inflammation.

Concurrent or co-morbid illness is a major limiting factor to tissue healing. As such, chronic wounds are uncommon in children. Therefore, paediatric patients with such complaint should be thoroughly investigated.

Management of chronic wounds depends largely on their aetiology. Following initial history and examination, imaging should be employed to further investigate the lesion. In young children ultrasound is the modality of choice, as it is non-invasive and better tolerated in a conscious patient. In most cases a combination of examination and radiological tests will point towards the cause of impaired healing.

When considering treatment, wounds secondary to foreign material require surgical excision in the vast majority of cases; nonetheless, cases of spontaneous resolution have been reported [9]. Debridement of necrotic material and foreign body excision remove the stimulant and tissues trapped in the inflammatory cycle, therefore, allowing the process of healing to restart. Superimposed infection should be eradicated with topical or systemic antimicrobials. In the paediatric population prognosis is good and complications are not normallyseen.

In conclusion, following review of invasive procedures associated with ECMO in this case, it is unclear as to how the stitch, resulting in chronic wound formation, was retained. It is possible that the small size of the patient and the large size of the ECMO cannulas contributed to the material sliding in between the tissue planes and being missed.

This case has clearly demonstrated that it is fundamental to investigated paediatric wounds that failed to heal after 6 to 12 weeks post-insult. In these cases, suture granulomas and retained foreign material should be suspected, especially in patients previously treated with indwelling intravascular cannulas.
